# Comparative metabolomics reveals complex metabolic shifts associated with nitrogen-induced color development in mature pepper fruit

**DOI:** 10.3389/fpls.2024.1319680

**Published:** 2024-02-20

**Authors:** Lu Zhang, Fen Zhang, Xuanyi He, Yuehua Dong, Kai Sun, Shunli Liu, Xiaozhong Wang, Huaiyu Yang, Wei Zhang, Prakash Lakshmanan, Xinping Chen, Yan Deng

**Affiliations:** ^1^ Interdisciplinary Research Center for Agriculture Green Development in Yangtze River Basin, College of Resources and Environment, Southwest University, Chongqing, China; ^2^ Key Laboratory of Low-carbon Green Agriculture in Southwestern China, Ministry of Agriculture and Rural Affairs, Southwest University, Chongqing, China; ^3^ Key Laboratory of Efficient Utilization of Soil and Fertilizer Resources, Southwest University, Chongqing, China; ^4^ Key Laboratory of Sugarcane Biotechnology and Genetic Improvement (Guangxi), Ministry of Agriculture and Rural Affairs; Guangxi Key Laboratory of Sugarcane Genetic Improvement, Sugarcane Research Institute, Guangxi Academy of Agricultural Sciences, Nanning, China; ^5^ Queensland Alliance for Agriculture and Food Innovation, The University of Queensland, Brisbane, QLD, Australia

**Keywords:** pepper, fruit color, nitrogen, carotenoids, flavonoids, metabolome

## Abstract

Pigments derived from red pepper fruits are widely used in food and cosmetics as natural colorants. Nitrogen (N) is a key nutrient affecting plant growth and metabolism; however, its regulation of color-related metabolites in pepper fruit has not been fully elucidated. This study analyzed the effects of N supply (0, 250, and 400 kg N ha^-1^) on the growth, fruit skin color, and targeted and non-target secondary metabolites of field-grown pepper fruits at the mature red stage. Overall, 16 carotenoids were detected, of which capsanthin, zeaxanthin, and capsorubin were the dominant ones. N application at 250 kg ha^-1^ dramatically increased contents of red pigment capsanthin, yellow-orange zeaxanthin and β-carotene, with optimum fruit yield. A total of 290 secondary metabolites were detected and identified. The relative content of most flavonoids and phenolic acids was decreased with increasing N supply. Correlation analysis showed that color parameters were highly correlated with N application rates, carotenoids, flavonoids, phenolic acids, lignans, and coumarins. Collectively, N promoted carotenoid biosynthesis but downregulated phenylpropanoid and flavonoid biosynthesis, which together determined the spectrum of red color expression in pepper fruit. Our results provide a better understanding of the impact of N nutrition on pepper fruit color formation and related physiology, and identification of target metabolites for enhancement of nutritional quality and consumer appeal.

## 1 Introduction

Chili peppers (*Capsicum annuum* L.) are cultivated and consumed worldwide. Approximately 38.03 million tons of fresh peppers and 4.25 million tons of dry peppers were produced globally in 2019 (Barik et al., 2022). Pepper fruit is a rich source of phytochemicals with antioxidant properties, such as carotenoids and flavonoids, and it is also used as natural colorant (del Rocío Gomez-Garcia and Ochoa-Alejo, 2013; Baenas et al., 2019). As such, the global demand for pepper fruit pigments for food and cosmetics is steadily increasing (Leong et al., 2018; Saini et al., 2022).

Carotenoids are tetraterpenoid pigments, and their chromogenic characteristics are usually what give the fruits their yellow, orange, red, or purple coloring (Maoka, 2020). Capsanthin and capsorubin (very pepper-specific pigments), which represent 40%–60% of the total carotenoids, confer pepper fruits their red color, while α-carotene, β-carotene, and zeaxanthin make yellow-orange-colored peppers (Baenas et al., 2019; Villa-Rivera and Ochoa-Alejo, 2020). Owing to their strong antioxidant properties, carotenoids are now widely used in food and cosmetics (Baenas et al., 2019). The global market for carotenoids was estimated to be $2.0 billion in 2022, with consumption reaching 6222.6 metric tons (Bogacz-Radomska et al., 2020), and capsanthin having the largest market share.

Flavonoids, together with phenolic acid derivatives, represent the major groups of phenolic compounds in pepper fruit, providing taste, color, and flavor to fruits (Shen et al., 2018). As carotenoids, flavonoids are among the most common and important sources of color pigments in plants, which can produce a spectrum of color from bright yellow to red or from faint yellow to orange (Scarano et al., 2018; Peng et al., 2022). Importantly, flavonol glycosides (such as quercetin-O-glycosides) are abundant in peppers and demonstrate higher levels of antioxidant and anti-inflammatory activity than other phenolic compounds (Barik et al., 2022).

Most of the previous studies on pepper fruit color have focused on individual or a select group of carotenoids or flavonoids (Dubey et al., 2015; Liu et al., 2020). Most studies report the characterization, quantitation, or genotypic distribution of carotenoids, total phenols, and flavonoids, and their antioxidant properties (Giuffrida et al., 2013; Wahyuni et al., 2013; Mi et al., 2020; Zoccali et al., 2021; Mi et al., 2022). Non-saponification is the common method used to differentiate carotenoids in these studies. For example, Giuffrida et al. (2013) identified 52 carotenoids by non-saponification and found considerable compositional variation among them in 12 different *Capsicum* cultivars. However, saponification employed to hydrolyze esters can simplify the chromatographic analysis of carotenoids, making it useful for the detection of minor carotenoids (Feng et al., 2022). Therefore, targeted carotenoid metabolomics using saponification has the potential to expand our knowledge of specific classes of carotenoid metabolites contributing to color expression. Additionally, non-targeted metabolomics can help to identify a broader spectrum of metabolic products and understand their metabolism in relation to fruit color development (Luo et al., 2022; Marinov et al., 2023). Until now, such comprehensive saponification-assisted targeted carotenoid metabolomics and non-targeted secondary metabolomics studies on pepper fruit color development have not been reported.

Besides the genotype, the diversity and content of carotenoids and flavonoids are influenced by crop environmental conditions, including nutrition. Nitrogen (N), amongst the main environmental factors, is one of the most important nutrients for crop growth, product quality, and yield formation (Farneselli et al., 2018; Zhao et al., 2021). It provides functional groups for carbon skeletons and modulates the biosynthesis of secondary metabolites, such as flavonoids, phenolic acids, and carotenoids (Narvekar and Tharayil, 2021). Generally, N supply has a negative effect on flavonoids and phenolic acids, whereas it increases the content of chlorophyll and carotenoids across different plant species (Ibrahim et al., 2012; Narvekar and Tharayil, 2021). However, the production of other phenolic compounds may be stimulated, or not affected with the increase in N supply (Bustamante et al., 2020; Saloner and Bernstein, 2021). Although N fertilization is a common practice used to increase yield in pepper production, research on the influence of N supply on pigment-related metabolites in pepper is rare, and much less is known about N-mediated metabolic regulation of pepper color development.

In the present study, we integrated targeted carotenoid metabolomics, non-targeted secondary metabolomics, and different color parameters for a comprehensive investigation of pepper fruit color formation as influenced by different N supply at the mature red stage. The results we report here will provide new insights into the N regulation of pigment production, and help specify potential targets for controlling pepper fruit color formation through molecular genetics and/or agronomic practices.

## 2 Materials and methods

### 2.1 Experimental site and treatments

This study was conducted in 2021 at the Hechuan Experiment Station of Southwest University in Weituo Town, Chongqing City, China (30°01′N, 106°13′E). This region has a typical subtropical monsoon climate. During the pepper season in 2021, the average air temperature and total precipitation were 26.5°C and 699 mm, respectively. A long-term field experiment on Chinese cabbage-pepper rotation was started in 2018, and the present work was carried out during the fourth pepper cropping season. The soil type was alluvium, and the chemical properties of the top 20-cm layer at the beginning of the long-term experiment were as follows: pH, 5.65 (soil-water ratio 1:2.5); organic matter, 9.21 g kg^-1^; total N, 0.50 g kg^-1^; available phosphorus (Olsen-P), 19.51 mg kg^-1^; and ammonium acetate extractable potassium (K), 56.0 mg kg^-1^.

The red chili pepper variety “Xinxiang 8” was planted under three N rate treatments: (1) control without N fertilizer (N0; 0 kg N ha^-1^, 140 kg P_2_O_5_ ha^-1^, 300 kg K_2_O ha^-1^); (2) recommended N rate based on root zone N management (N250; 250 kg N ha^-1^, 140 kg P_2_O_5_ ha^-1^, 300 kg K_2_O ha^-1^); and (3) N rate routinely used by farmers (N400; 400 kg N ha^-1^, 290 kg P_2_O_5_ ha^-1^, 230 kg K_2_O ha^-1^). The N400 treatment was set based on the fertilization practice found in a survey of 314 farmers conducted as part of our preliminary work, which found that inputs of N and P were too high but input of K was relatively low. Therefore, the N0 and N250 treatments were set based on optimizing P and K input to meet the needs of pepper growth. The three treatments were arranged in a randomized complete block design with four replicates, totaling 12 plots. Each plot (replicate) measured 46.6 m^2^ (8.25 m × 5.65 m), and they were spaced 1.5 m apart. A total of 160 plants were grown in rows (0.6-m row spacing) with 0.4-m inter-plant distance in each plot. Chemical fertilizers urea (46% N), superphosphate (12% P_2_O_5_), and potassium sulphate (50% K_2_O) were used to supply the N, P, and K nutrients. The amount of fertilizer and application date for different growth periods are presented in **Supplementary Table S1**. The experiment was conducted from 20 May to 1 September, 2021, and the crop management followed the local conventional agronomy practices for pepper production.

### 2.2 Plant and soil sampling

Pepper fruits were sampled at the mature red stage (16 August, 2021) (**Supplementary Figure S1**). In each plot, 10 plants of similar growth were randomly selected, and fruits, two per plant, were collected from the same plant part of the chosen plants. From each plot (replicate), half of the sample (10 fruits) was used for measuring single fruit fresh weight and surface color parameters, and the remaining half (10 fruits) was quickly cleaned and dissected. Pericarps were then collected and combined into one replicate sample. This sampling procedure was followed to produce four independent replicate samples for each treatment. Homogenized pericarp samples were frozen immediately in liquid nitrogen, transported to the laboratory and stored at -80°C for metabolite analysis. Simultaneously, soil samples from the top 20-cm layer were taken from each plot for measurement of soil mineral N (NH_4_^+^-N and NO_3_^–^N) level.

### 2.3 Soil mineral N level analysis

In the laboratory, fresh soil samples from the top 20-cm layer were quickly sieved (2-mm sieve size), then sub-samples were taken and extracted by 0.01 mol L^-1^ CaCl_2_ solution. Meanwhile, sub-samples were oven-dried to measure soil water content. The NH_4_^+^-N and NO_3_^–^N content in the extraction solution was determined using an Auto Analyzer 3 Continuous-flow Analysis-CFA (SEAL Analytical GmbH, Norderstedt, Germany). Soil NH_4_^+^-N and NO_3_^–^N level was calculated based on soil dry weight.

### 2.4 Determination of pepper fruit growth and nutrient content

For measurement of single fruit fresh weight, 10 pepper fruits per replicate sample were weighed individually, and the average weight was calculated for each treatment replicate. To determine fruit yield and biomass, 24 (six plants × four rows) plants were selected from the middle of each plot, and all the fruits from the chosen plants were picked. Their fresh weight was measured and then dried at 75°C to measure dry weight. More specially, after drying, pericarp and the other parts were separated and weighed individually. Fruit yield and fruit biomass per hectare were calculated based on sampling area and fruit fresh weight (yield) or dry weight (biomass). Dried fruit samples were finely powdered and used for N, P, and K measurement. Fruit N content was determined using the Kjeldahl method (Yang et al., 2008), while P and K were assayed using inductively-coupled plasma-optical emission spectrometry (ICP-OES, 5110 SVDV; Agilent, Santa Clara, CA, United States) (Lu et al., 2021).

### 2.5 Measurement of fruit surface color

Surface color of harvested pepper fruits was determined using a colorimeter (CR-10Plus; Konica Minolta, Tokyo, Japan). After measuring the single fruit fresh weight, the same 10 fruits were used for fruit color measurement for each treatment replicate, with each fruit sample measured at three different positions. The CIE (International Commission on Illumination) color parameters, i.e., L* value (brightness), a* (green-red index), and b* (yellow-blue index) coordinates were used to describe the color. The hue angle [h* = tan^-1^(b*/a*)] (when h* < 50, the smaller, the redder) and chroma [C* = (a*^2^ + b*^2^)/2)] parameters were calculated as reported previously (Guo et al., 2021; Feng et al., 2022).

### 2.6 Determination of total phenol content

The total phenol content in pericarp samples was determined by the Folin–Denis method using assay kits (Suzhou Grace Biotechnology Co., Ltd., Suzhou, China) according to the manufacturer’s instructions. Briefly, 0.2 g of ground sample was mixed with 1.5 mL of 60% ethanol, then the mixture was stirred for 1 h at 60°C, and centrifuged at 12,000 rpm under 25°C for 10 min. The supernatant was collected and its volume was adjusted to 1.5 mL with 60% ethanol. This ethanolic extract was assayed for phenols using the assay kit. The total phenol content was determined at 760 nm with a UV-5200 spectrophotometer (Shanghai Metash Instruments Co., Ltd., Shanghai, China), and the content was expressed on sample fresh weight basis (mg g^-1^ FW).

### 2.7 Carotenoid identification and quantification

The extraction of carotenoids by saponification and the analysis of targeted carotenoids were performed by Metware Biotechnology Co., Ltd., Wuhan, China (http://www.metware.cn), according to the procedure described by Feng et al. (2022) with some modifications. More specifically, 50 mg freeze-dried pericarp powder was vortexed with 0.5 mL n-hexane/acetone/ethanol (1:1:1, v/v/v) solution for 20 min at room temperature, then the mixture was centrifuged at 12,000 rpm for 5 min at 4°C and the supernatant was collected. This extraction step was repeated once. The combined supernatant was mixed with 0.5 mL of saturated NaCl solution and vortexed until the solution was stratified, and the supernatant was collected. This step was repeated twice. Afterwards, the supernatant was evaporated to dryness and the dry residue was dissolved in 0.5 mL of methyl tert-butyl ether (MTBE), and saponified with 0.5 mL of 10% KOH-MeOH for 18 h in the dark at room temperature. The saponified extract was thoroughly mixed with 1 mL of saturated NaCl solution and 0.5 mL of MTBE and the upper layer was collected. This step was repeated twice. The combined supernatant was evaporated to dryness and reconstituted with 100 μL of MeOH/MTBE (1:1, v/v) solution. The solution was filtered through a 0.22-μm membrane filter and used for identification and quantification of carotenoids using an LC-APCI-MS/MS system (UPLC: ExionLC™ AD, Sciex Framingham, MA, USA; MS: 6500 Triple Quadrupole, Applied Biosystems, Foster City, CA, USA). The working conditions and procedures of this analysis system are described in **Supplementary Method S1**.

### 2.7 Non-targeted secondary metabolite analysis

Non-targeted metabolome analysis was also performed by MetWare Biotechnology Co., Ltd., Wuhan, China. The pericarp samples were freeze-dried in a vacuum freeze-dryer (Scientz-100F; Scientz, Ningbo, China) and then crushed using a mixer mill (MM 400; Retsch, Haan, Germany) with zirconia beads for 1.5 min at 30 Hz. The lyophilized powder (100 mg) was dissolved in 1.2 mL of 70% methanol solution, vortexed for 30 s every 30 min for six times, and stored in a refrigerator at 4°C overnight. The next day, the mixed solution was centrifugated at 12,000 rpm for 10 min and the supernatant was filtrated (SCAA-104, 0.22-μm pore size; ANPEL, Shanghai, China, http://www.anpel.com.cn/). All extracts were analyzed using an UPLC-ESI-MS/MS system (UPLC: Nexera X2, SHIMADZU, Kanagawa, Japan; MS: 4500 Q TRAP, Applied Biosystems). The working conditions and procedures of this analysis system are described in **Supplementary Method S2**.

Metabolites in the extracts were identified by comparing their spectral information to the standard reference materials in the MWDB database (MetWare Biological Science and Technology Co., Ltd.). They were quantified by triple quadrupole mass spectrometry using multi-reaction monitoring (MRM) mode. To produce maximal signal, collision energy and de-clustering potential were optimized for each precursor-product ion (Q1–Q3) transition (Zhu et al., 2018). The chromatographic peak area integral was used to represent the metabolite content. The potential differentially-accumulated metabolites (DAMs) between pairs of N treatments were identified using VIP (variable importance in projection) ≥ 1 criterion in the orthogonal partial least squares-discriminant analysis (OPLS-DA) results. A permutation test with 200 iterations was performed. Next, the DAMs between N treatments were identified based on VIP ≥ 1 and *P* < 0.05 (t-test). Identified metabolites were further annotated using the Kyoto Encyclopedia of Genes and Genomes (KEGG) compound database (http://www.kegg.jp/kegg/compound/) and mapped using the KEGG pathway database (http://www.kegg.jp/kegg/pathway.html). Pathways with corrected *P*-values ≤ 0.05 (hypergeometric test) were considered significantly altered. The carotenoid, phenylpropanoid, and flavonoid biosynthesis pathways were constructed based on the KEGG pathway and published literature (Tohge et al., 2017; Berry et al., 2019; Villa-Rivera and Ochoa-Alejo, 2020).

### 2.8 Data analysis

Statistical analyses were performed using SPSS 25.0 (SPSS, Inc., Chicago, IL, USA). Data were subjected to one-way ANOVA and significant differences were analyzed by the Duncan’s multiple range test at *P* < 0.05. The cluster heat map (normalized by Z-score) was performed using Origin 2021 (OriginLab Corp., Northampton, MA, United States). Correlation coefficients were determined by the Pearson test. Correlations with a coefficient (r) value > 0.7 (positive) or < −0.7 (negative) were considered to be crucial relationships. The relationship visualization was performed using Cytoscape software (version 3.9.1; https://apps.cytoscape.org/).

## 3 Results

### 3.1 Soil mineral N level, pepper fruit growth, and nutrient content

**Figure 1:**
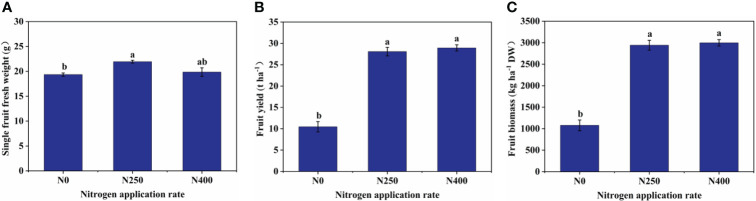
Single fruit fresh weight **(A)**, fruit yield **(B)**, and fruit biomass **(C)** of mature red peppers produced under different N application rates. Values are presented as means ± SE (n = 4). Different lowercase letters above the bars indicate a significant difference between N application rates at a *P* < 0.05 level (Duncan’s test).

At harvest, with increasing N supply, soil NH_4_^+^-N levels did not differ much between treatments while soil NO_3_^–^N levels were greatly increased under N250 and N400 conditions (**Supplementary Table S2**). Correspondingly, single fruit fresh weight, fruit yield, fruit biomass (dry weight), and fruit N content were all significantly increased under N250 treatment compared to the control condition (N0) (**Figure 1**; **Supplementary Table S2**). No further change in any of these parameters occurred with increased N supply (N400). On the other hand, N treatments did not change fruit P and K content significantly (**Supplementary Table S2**).

### 3.2 Color characteristics of pepper fruit

**Figure 2:**
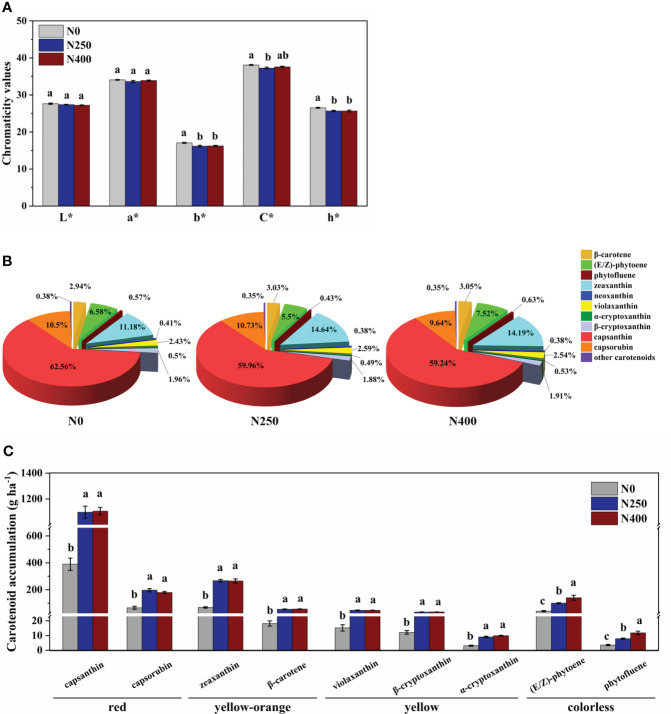
Color parameters **(A)**, proportion of different carotenoids in total carotenoid content **(B)**, and accumulation per hectare of main carotenoids **(C)** for mature red pepper fruits produced under different N application rates. L*, brightness index; a*, green-red index; b*, yellow-blue index; h*, hue angle, h* = tan^-1^(b*/a*); C*, chroma index, C* = (a*^2^ + b*^2^)/2. In plot **(A, C)**, values are presented as means ± SE (n = 4), and different lowercase letters above the bars indicate a significant difference between N application rates at a *P* < 0.05 level (Duncan’s test).

The L* (brightness) and a* (green-red index) values of fruit skin were not affected by N application rate. However, compared to N0 treatment, the b* (yellow-blue index), C* (chroma), and h* (hue angle) values were decreased with N application (N250 and N400 conditions), with no significant difference detected between the two N treatments (**Figure 2A**).

### 3.3 Carotenoids and total phenols in pepper fruit

For carotenoid identification, a typical total ion chromatogram (TIC) of a quality control (QC) sample is shown in **Supplementary Figure S2A**, with each differently-colored peak representing a detected metabolite. A multi-peak detection plot of metabolites acquired in multiple reaction monitoring (MRM) mode is shown in **Supplementary Figure S2B**. Following such measuring standards, a total of 20 carotenoids were detected in pepper fruit pericarp, but only 16 were identified, with the other four detected at a concentration too low to be identified (**Supplementary Table S3**).

The 16 identified carotenoids in pericarp included six carotenes and 10 xanthophylls (**Table 1**). With increasing N supply, no consistent trend in the content of different carotenoids was observed. The content of total carotenes in fruits produced under N0 and N250 treatments was similar, but was significantly higher in those produced under N400. The total xanthophyll content increased remarkably with N application (N250 and N400). As a result, the response of total carotenoid content to N supply was similar to that of total xanthophyll. In contrast to carotenoids, the total phenol content was significantly reduced with N application (N250 and N400), but with no N rate effect.

**Table 1:** Content of carotenoids and total phenols in the pericarp of mature red pepper fruit produced under different N application rates.

Compounds		Coloration	N application rate		
			N0	N250	N400
Carotenes (μg g^-1^ DW)	α-carotene	yellow-orange	2.01 ± 0.06**^a^**	2.04 ± 0.08**^a^**	2.04 ± 0.08**^a^**
	β-carotene	yellow-orange	23.27 ± 0.42**^b^**	25.84 ± 0.56**^a^**	26.03 ± 0.90**^a^**
	phytofluene	colorlesss	4.51 ± 0.18**^ab^**	3.68 ± 0.2**^b^**	5.4 ± 0.52**^a^**
	(E/Z)-phytoene	colorlesss	52.1 ± 0.68**^ab^**	46.85 ± 1.45**^b^**	64.12 ± 7.80**^a^**
	γ-carotene	yellow	0.29 ± 0.01**^a^**	0.26 ± 0.005**^b^**	0.25 ± 0.01**^b^**
	ϵ-carotene		0.13 ± 0.002**^b^**	0.14 ± 0.006**^ab^**	0.16 ± 0.015**^a^**
	Total carotenes		82.31 ± 1.14**^b^**	78.8 ± 1.95**^b^**	98.01 ± 7.74**^a^**
Xanthophylls (μg g^-1^ DW)	capsanthin	intense red	495.55 ± 2.51**^b^**	510.45 ± 2.65**^a^**	505.11 ± 5.88**^ab^**
	capsorubin	red-brown	83.17 ± 3.35**^a^**	91.42 ± 3.3**^a^**	82.23 ± 4.84**^a^**
	zeaxanthin	yellow-orange	88.55 ± 3.64**^b^**	124.7 ± 4.73**^a^**	120.99 ± 6.81**^a^**
	violaxanthin	yellow	19.24 ± 0.48**^b^**	22.07 ± 0.50**^a^**	21.65 ± 0.62**^a^**
	neoxanthin	yellow	3.21 ± 0.06**^a^**	3.22 ± 0.18**^a^**	3.23 ± 0.40**^a^**
	β-cryptoxanthin	yellow	15.54 ± 0.43**^a^**	16.05 ± 0.68**^a^**	16.27 ± 0.52**^a^**
	α-cryptoxanthin	yellow	3.98 ± 0.09**^b^**	4.21 ± 0.09**^b^**	4.54 ± 0.13**^a^**
	echinenone		0.02 ± 0.0005**^a^**	0.02 ± 0.0006**^a^**	0.02 ± 0.001**^a^**
	β-citraurin		0.48 ± 0.01**^a^**	0.42 ± 0.01**^a^**	0.45 ± 0.03**^a^**
	8’-apo-beta-carotenal		0.07 ± 0.002**^b^**	0.10 ± 0.004**^a^**	0.10 ± 0.003**^a^**
	Total xanthophylls		709.81 ± 1.88**^b^**	772.66 ± 5.18**^a^**	754.59 ± 8.82**^a^**
Total carotenoids (μg g^-1^ DW)			792.12 ± 1.03**^b^**	851.46 ± 6.93**^a^**	852.6 ± 4.04**^a^**
Total phenols (mg g^-1^ FW)			1.24 ± 0.03**^a^**	1.00 ± 0.03**^b^**	1.05 ± 0.06**^b^**

In the same row values (mean ± SE, n = 4) followed by different superscript letters indicate a significant difference between N application rates at a P < 0.05 level (Duncan’s test).

Capsanthin, zeaxanthin, capsorubin and (E/Z)-phytoene were the main carotenoids detected in pepper pericarp (**Table 1**). Together, they accounted for approximately 90% of the total carotenoid content, with capsanthin being the most dominant carotenoid (approximately 60%) (**Figure 2B**). N application significantly decreased the proportion of capsanthin in total carotenoid content, while an opposite trend was evident for zeaxanthin (**Figure 2B**). Based on the tissue content and pericarp biomass (dry weight), the accumulation of main carotenoids per hectare was calculated and the results are presented in **Figure 2C**. The accumulation of red (capsanthin and capsorubin), yellow-orange (zeaxanthin and β-carotene), and yellow (violaxanthin, β-cryptoxanthin and α-cryptoxanthin) pigments were increased significantly with N application (N250 and N400), but with no significant N rate effect. However, the accumulation of colorless pigments, (E/Z)-phytoene and phytofluene, kept increasing with the increase in N application rate.

### 3.4 Carotenoid biosynthesis pathway analysis

**Figure 3:**
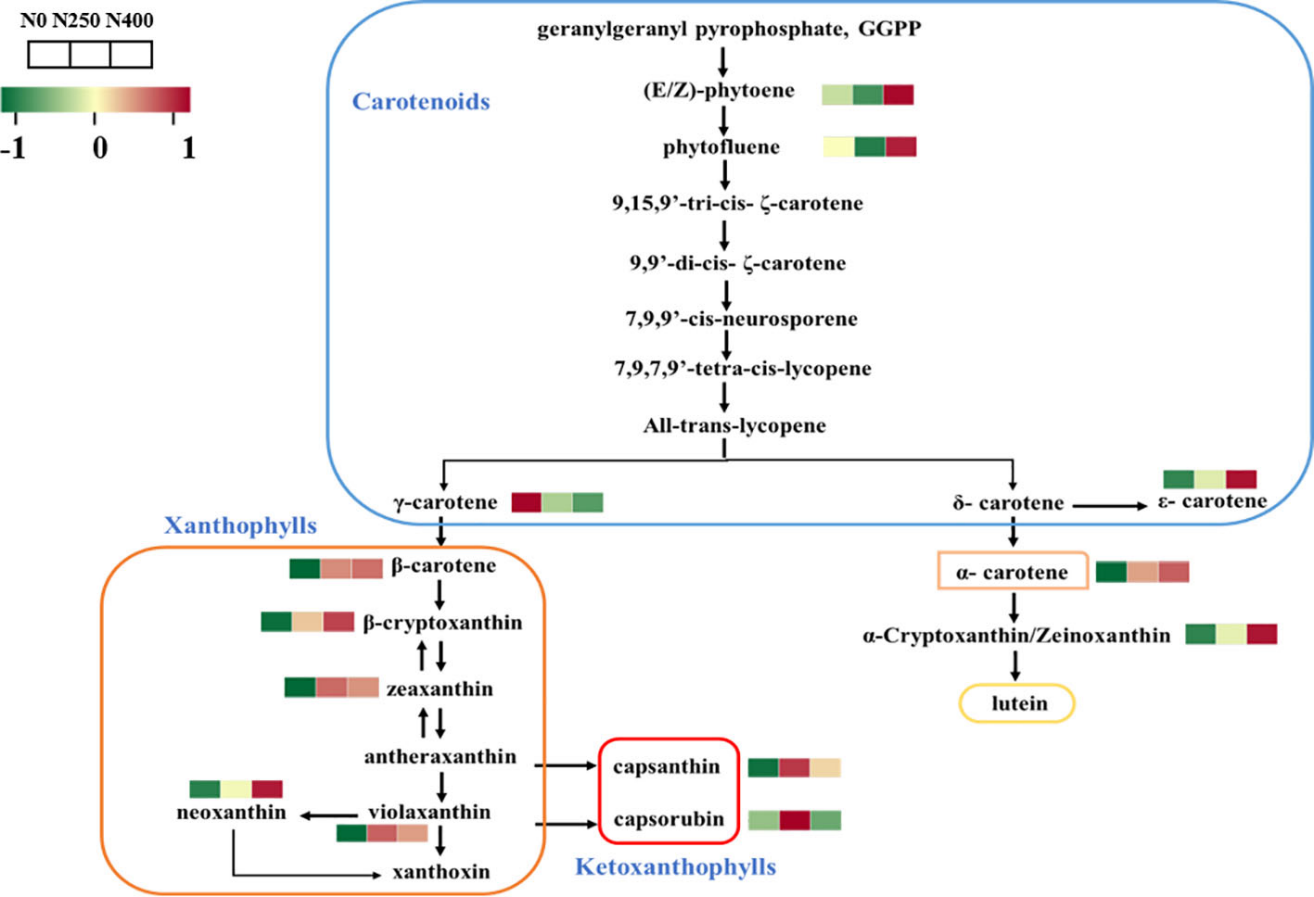
Heat map of metabolites involved in carotenoid biosynthesis following different N application rates. Each colored cell represents the normalized content of each compound by standardized Z-score (mean of four replicates for each N rate). The Z score is shown on a green (low) to red (high) color scale. As relative content of metabolites increases, the color of the cell changes from green to red.

The carotenoid biosynthesis pathway in pepper was constructed based on the KEGG pathway and literature references (**Figure 3**). Thirteen of the 16 identified carotenoids were mapped in the pathway. As the primary carotenoid metabolites, colorless pigments (E/Z)-phytoene and phytofluene were reduced under N250 treatment but were increased greatly under N400 treatment compared with the control condition (N0). A similar increasing trend in response to N supply was found for carotenoid pigments in the δ-carotene pathway. Nitrogen application decreased the content of γ-carotene and increased the level of its derivatives in the γ-carotene pathway. Capsanthin and capsorubin, the most abundant xanthophyll derivatives, were increased remarkably in fruits produced under N250 compared with those grown under the control (N0) and high N (N400) conditions.

### 3.5 Identification and analysis of secondary metabolites

A total of 290 secondary metabolites, including 133 flavonoids, 135 phenolic acids, 12 lignans and 10 coumarins were identified. OPLS-DA analysis helped maximize the identification of metabolites with little quantitative difference between them, which aided screening for metabolites with differential accumulation (**Supplementary Figure S3**). R^2^Y scores and Q^2^ values represent the interpretation rate of the OPLS-DA model to the Y matrix and the prediction ability of the model, respectively. Results showed that R^2^Y scores were all higher than 0.99, and Q^2^ values were all larger than 0.68, confirming the differential accumulation response of metabolites to N treatment (**Supplementary Figure S4**).

**Figure 4:**
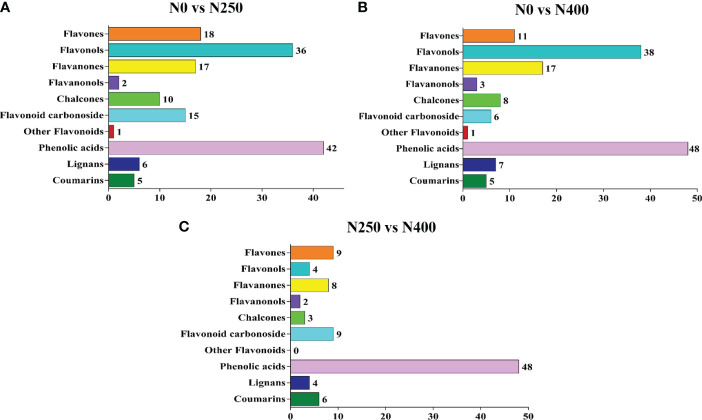
The number of potential differentially-accumulated metabolites in the pericarp of mature red pepper fruits in the following comparisons: N0 vs. N250 **(A)**, N0 vs. N400 **(B)**, and N250 vs. N400 **(C)**. The metabolites are screened according to variable importance in projection (VIP) ≥ 1 in the orthogonal partial least squares-discriminant analysis (OPLS-DA).

Potential DAMs were identified using the VIP ≥ 1 criterion. A total of 152, 144, and 93 potential DAMs were identified from N0 vs. N250, N0 vs. N400, and N250 vs. N400 comparisons, respectively (**Figure 4**; **Supplementary Table S4**). From this analysis, N0 vs. N250 (**Figure 4A**; **Supplementary Table S4A**) and N0 vs. N400 (**Figure 4B**; **Supplementary Table S4B**) comparisons had the greatest number of flavonoids (especially flavonols, flavanones, and flavones) and phenolic acids. In the N250 vs. N400 comparison, most potential DAMs were phenolic acids (**Figure 4C**; **Supplementary Table S4C**).

**Figure 5:**
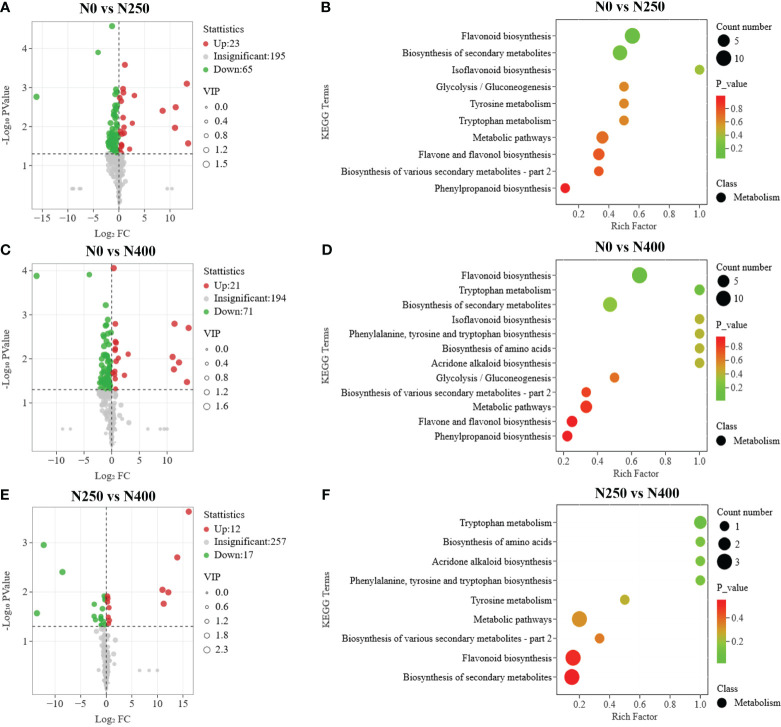
Differential metabolite analysis and Kyoto Encyclopedia of Genes and Genomes (KEGG) enrichment analysis for comparisons of N0 vs. N250 **(A, B)**, N0 vs. N400 **(C, D)**, and N250 vs. N400 **(E, F)**. In the volcano plot **(A, C, E)**, the red dots represent upregulated metabolites, blue dots represent downregulated metabolites and gray dots represent metabolites with no significant difference. In the KEGG enrichment plot **(B, D, F)**, each circle represents the number of associated metabolites and is positioned according to its enrichment factor. The *P*-values represent the hypergeometric test result of the degree of differential metabolite enrichment, which are indicated by color scale from green (low) to red (high).

To further screen for the DAMs occurring in different pairwise comparisons of N treatments, the DAMs were screened according to the combination of VIP ≥ 1 and *P* < 0.05 (t-test). The relationships between VIP value and *P*-value for different group comparisons were analyzed to confirm the validity of DAM identification (**Supplementary Figure S5**). The number of DAMs was 88 between N0 and N250 (23 up, 65 down) (**Figure 5A**; **Supplementary Table S5A**), 92 between N0 and N400 (21 up, 71 down) (**Figure 5C**; **Supplementary Table S5B**), and 29 between N250 and N400 (12 up, 17 down) (**Figure 5E**; **Supplementary Table S5C**). Notably, lignans (epipinoresinol and pinoresinol) were upregulated > 4-fold, while the flavonoids (luteolin-7-O-neohesperidoside, kaempferol-3-O-glucorhamnoside, kaempferol-3-O-neohesperidoside, kaempferol-3-O-rutinoside, 6-C-glucosyl-2-hydroxynaringenin, eriodictyol-8-C-glucoside, choerospondin, aromadendrin-7-O-glucoside, and dihydrocharcone-4′-O-glucoside) and phenolic acids (6-O-caffeoylarbutin and 3-O-p-Coumaroylquinic acid-O-glucoside) were decreased < 0.3-fold under N250 and N400 conditions compared with the control (N0) condition (**Supplementary Table S5**).

Additionally, eriodictyol-7-O-glucoside (a flavanone), decursinol and acoumarin were only detected in N250 and N400, and apigenin-6-C-(2″-xylosyl) glucoside, a flavonoid carbonoside, was found only in N0 and N400. The flavanones 5,4′-dihydroxy-7-methoxyflavanone (sakuranetin) and persicoside, and the chalcones 2′,3,4,4′,6′-pentahydroxychalcone-4′-O-glucoside were detected only in N250, while dihydromyricetin, aflavanonol, and the phenolic acids 2-hydroxybenzaldehyde, anthranilic acid, and trihydroxycinnamoylquinic acid were identified in the N400 condition only (**Supplementary Tables S4**, **S5**).

To identify the trends between DAM content and N application rate, K-means analysis was performed (**Supplementary Figure S6**). The 129 DAMs were further divided into six sub-classes (1 to 6), containing 60, 20, 11, 18, 12, and 6 metabolites, respectively (**Supplementary Figure S6**; **Supplementary Table S6**). The DAMs in sub-class 1, 2, and 6 were mainly flavonoids and phenolic acids, while those in sub-class 3, 4, and 5 were mostly lignans and coumarins, with some flavonoids and phenolic acids (**Supplementary Table S6**). The DAM content in sub-class 1, 2, and 6 were decreased with N supply (N250) (**Supplementary Figures S6A, B, F**), while those in sub-class 3, 4, and 5 were increased with the same N treatment (N250) (**Supplementary Figures S6C-E**). When N supply was further increased to N400, changes in metabolite content displayed a downward trend for DAMs in sub-class 2, 3, and 5, whereas it was increased for those in sub-class 4 and 6, and remained stable for DAMs in sub-class 1.

### 3.6 Metabolic pathway enrichment analysis

The pathways associated with pigment metabolites were identified using the KEGG database. The number of DAMs annotated by KEGG with significant difference from the N0 vs. N250, N0 vs. N400, N250 vs. N400 comparisons was 19, 20, and 7, respectively (**Figures 5B, D, F**). The most enriched KEGG terms among the DAMs were flavonoid biosynthesis, flavone and flavonol biosynthesis, and phenylpropanoid biosynthesis. The DAMs involved in the flavonoid biosynthesis pathway were highly enriched in the N0 vs. N250 and N0 vs. N400 comparisons.

**Figure 6:**
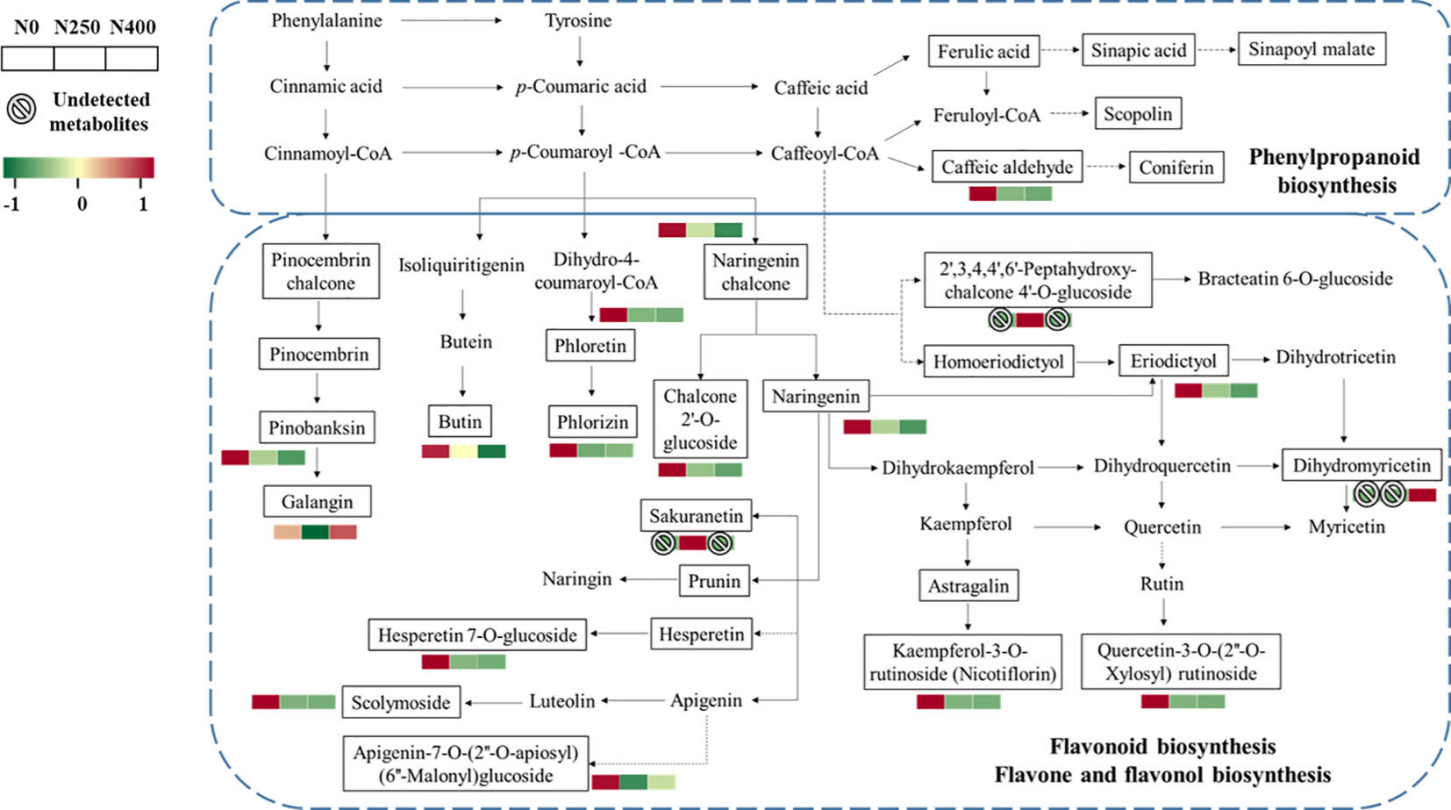
Heat map of metabolites involved in phenylpropanoid and flavonoid biosynthesis pathways as affected by different N application rates. Each colored cell represents the normalized accumulation of each compound by standardized Z-score (mean of four replicates for each N rate). The Z score is shown on a green (low) to red (high) color scale. As relative accumulation of metabolites increases, the color of the cell changes from green to red. The metabolites in the box and not shown in red or green scale represent those that are detected but do not change significantly in response to N supply.

More specifically, the main metabolites of flavonoid biosynthesis (naringenin, eriodictyol, hesperetin-7-O-glucoside, pinobanksin, naringenin chalcone, phloretin, and phlorizin), flavone and flavonol biosynthesis (luteolin-7-O-neohesperidoside, kaempferol-3-O-rutinoside [nicotiflorin], quercetin-3-O-[2″-O-xylosyl] rutinoside), and phenylpropanoid biosynthesis (caffeic aldehyde) were downregulated with N application (N250 and N400) (**Figure 6**). The flavanonol dihydromyricetin involved in flavonoid biosynthesis was only detected under N400 treatment, while flavanone metabolite sakuranetin (5,4′-dihydroxy-7-methoxyflavanone) and chalcone metabolite 2′,3,4,4′,6′-pentahy droxychalcone-4′-O-glucoside were found only under N250.

### 3.7 Relationship between color parameters and metabolites

**Figure 7:**
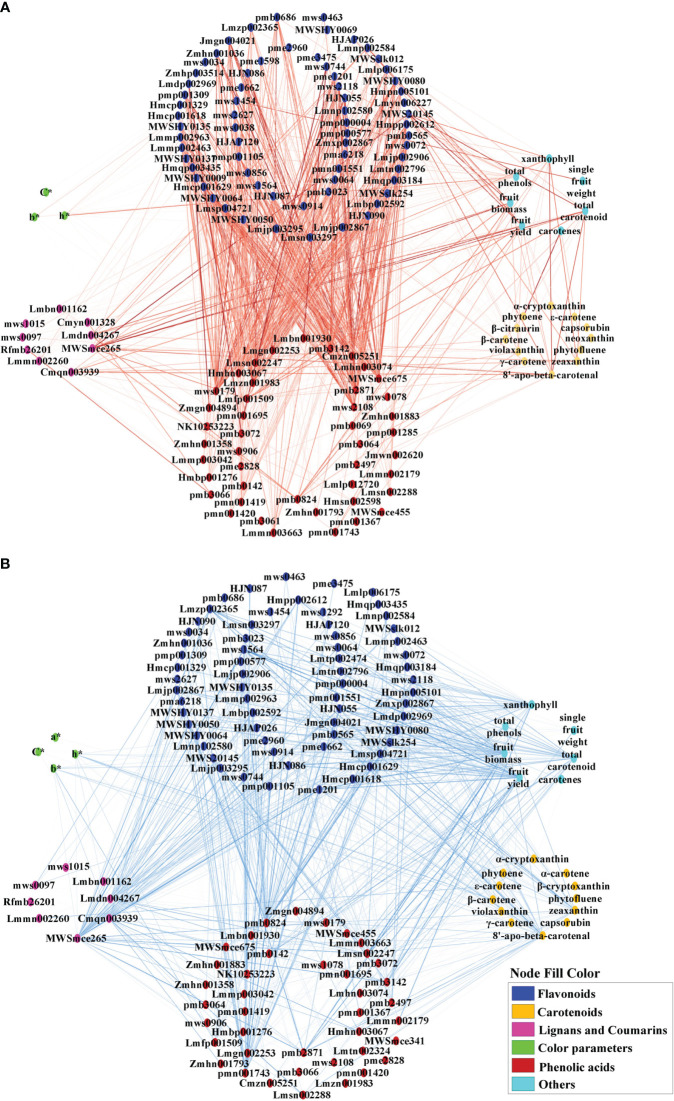
Positive **(A)** and negative **(B)** correlation network analysis of color parameters, growth indicators, pigments, and associated metabolites for mature red pepper fruit based on Pearson correlation. a*, green-red index; b*, yellow-blue index; h*, hue angle, h* = tan^-1^(b*/a*); C*, chroma index, C* = (a*^2^ + b*^2^)/2. Edges colored in red and blue represent positive and negative correlations, as determined by a Pearson correlation coefficient > 0.7 or < −0.7 (*P* < 0.05), respectively. The width of the edges indicates the strength of the correlation.

To investigate the correlations between metabolites and color parameters in pepper fruit, a correlation network analysis of color parameters, carotenoids, flavonoids, phenolic acids, lignans, and coumarins was conducted (**Figure 7**; **Supplementary Table S7**). Most carotenoids were negatively correlated with flavonoids and phenolic acids (**Figure 7B**). Most flavonoids were positively correlated with phenolic acids (**Figure 7A**) and negatively correlated with coumarin and lignans (**Figure 7B**). Total phenol content was negatively correlated with β-carotene, zeaxanthin, violaxanthin, fruit fresh weight and fruit yield (**Supplementary Table S7**). The N application rate showed a negative correlation with L*, b*, and h* values, total phenols, and most flavonoids and phenolic acids, but a positive correlation with most carotenoids, total carotenoids, lignans, coumarins, and yield (**Supplementary Table S7**).

The hue angle h* value showed a significant positive correlation with most flavonoids and phenolic acids, but was negatively correlated with capsanthin, capsorubin, β-carotene, zeaxanthin, violaxanthin, 8′-apo-beta-carotenal, total phenol, some ligans (liriodendrin, epipinoresinol, syringaresinol-4′-O-[6″-acetyl] glucoside) and coumarins (decursinol and esculin [6,7-dihydroxycoumarin-6-glucoside]) (**Supplementary Table S7**).

Capsanthin and capsorubin are unique pigments in pepper fruit. Capsanthin was negatively correlated with flavones (chrysoeriol-5,7-di-O-glucoside, luteolin-7-O-(6″-malonyl)glucoside-5-O-arabinoside, rhamnetin-3-O-rutinoside, quercetin-3-O-xylosyl(1→2) glucosyl (1→2)glucoside) and phenolic acid (3-O-p-Coumaroylquinic acid-O-glucoside), and positively correlated with flavonoid carbonoside (chrysoeriol-6-C-glucoside-4′-O-glucoside), lignan (pinoresinol), and coumarin (decursinol) (**Supplementary Table S7**).On the other hand, capsorubin was positively correlated with flavanones (persicoside), and negatively correlated with flavonoid carbonoside (apigenin-6-C-(2″-xylosyl) glucoside) and phenolic acid (protocatechuic acid-4-O-glucoside) (**Supplementary Table S7**).

## 4 Discussion

### 4.1 Reduced N supply is optimal for maximum pepper fruit yield and superior fruit quality

N is a vital nutrient required for plant growth and development. In this study, the three N rate treatments influenced pepper fruit N content but not P and K content (**Supplementary Table S2**), indicating that plant N nutrition is an important determinant of differential responses of pepper fruits grown under these N treatments. A highly positive correlation (*P* ≤ 0.01) between N application rate and fruit yield was observed (**Supplementary Table S7**). However, N supply beyond 250 kg ha^-1^ had little impact on fruit yield (**Figure 1**) and fruit N content (**Supplementary Table S2**). Thus, N supply at 250 kg ha^-1^ is considered optimal for maximum pepper yield. Moreover, a much lower level of soil NO_3_^–^N was found under N250 treatment than that under N400 treatment (**Supplementary Table S2**), indicating a lesser risk of NO_3_^–^N leaching and denitrification loss.

Fruit color is one of the criteria determining the commercial value of peppers (Korkmaz et al., 2021). Red pepper is generally evaluated by L*, a *, and b * color parameters (Baenas et al., 2019). Discolored red pepper skins have lower L* (brightness), a* (redness) and b* (yellowness) values than normal red pepper fruits (Feng et al., 2022). In this study, the L* and a* values were not affected by N application rate, whereas the b* value showed a significant reduction with N application (N250 and N400) compared with N0 supply (**Figure 2A**). As a result, the h* value (h* = tan^-1^(b*/a*) was substantially decreased with N supply (N250 and N400), making pepper fruits more reddish. Because there was no significant difference for these color parameters between N250 and N400, N application at 250 kg ha^-1^ is also sufficient to produce commercially-appealing red-colored pepper fruits. Thus, a much lower N input (250 kg N ha^-1^) than the current farmer practice (400 kg N ha^-1^) will produce more nutritious pepper without compromising yield and reduce fertilizer cost with potential environmental benefits.

### 4.2 Level of N supply regulates pepper fruit carotenoids content and composition

As a large class of natural lipid-soluble pigments, carotenoids may respond differently to N supply in different plants. Previous studies have reported positive effects of increased N application on β-carotene content in grapes and lutein in kiwiberry fruits (Gutierrez-Gamboa et al., 2018; Stefaniak et al., 2020). More than 50 carotenoids have been identified in different red pepper varieties (Arimboor et al., 2015), and the type and content of carotenoids are known to change with pepper fruit development (Liu et al., 2020). In this study, a total of 16 carotenoids were identified in the pericarp of red chili pepper at full maturity (**Table 1**). Of these, capsanthin, zeaxanthin, capsorubin, and (E/Z)-phytoene were the main compounds. The content of colored carotenoids capsanthin (intense red), zeaxanthin (yellow-orange), and capsorubin (red-brown) peaked with N application at 250 kg ha^-1^, while the colorless carotenoid, (E/Z)-phytoene, demonstrated its lowest level under this treatment. Capsanthin was the most dominant (approximately 60%) carotenoid in pepper fruit and its proportion was reduced with increased N supply, while the proportion of zeaxanthin, the second most abundant pigment, increased initially (N250) and remained unchanged with increased N supply (N400) (**Figure 2B**). The proportion of capsorubin and (E/Z)-phytoene in the total carotenoid content responded differently with changing N application rate. However, when calculated on a hectare basis using tissue (pericarp) content and tissue dry weight, the amount of all main carotenoids was increased significantly with N supply, with no difference detected between the N250 and N400 conditions except for the N rate-dependent rise in (E/Z)-phytoene accumulation (**Figure 2C**). Thus, the variation in response of tissue carotenoid content and carotenoid production in pepper per unit cropped area was due to the differential response of carotenoid levels in the tissue and fruit yield to the N application rate (**Table 1**; **Figures 1B, C**).

The content and composition of the carotenoids in pepper fruits during ripening are determined by two metabolic processes, i.e., transformation of existing photosynthetic pigments and *de novo* carotenoid biosynthesis (del Rocío Gomez-Garcia and Ochoa-Alejo, 2013; Berry et al., 2019; Zheng et al., 2019). The first committed step for carotenoid biosynthesis involves the condensation of two geranylgeranyl diphosphate molecules into colorless phytoene catalyzed by phytoene synthase (PSY) (Meng et al., 2019). Then, the synthesis of carotenoid is divided into two branches mediated by two key enzymes, lycopene β-cyclase (LCYB) and lycopene ϵ-cyclase (LCYE) (Liu et al., 2020). Finally, capsanthin-capsorubin synthase (CCS) transforms zeaxanthin and violaxanthin into capsanthin and capsorubin respectively, and the final products of the carotenoid biosynthetic pathway produce the red color of pepper fruits (Guo et al., 2021). In this study, 13 out of the 16 identified carotenoids were related to the *de novo* carotenoid biosynthesis (**Figure 3**). The KEGG pathway mapping showed that the effects of different N supply on the accumulation of primary precursors, (E/Z)-phytoene and phytofluene, differed from that on the downstream carotenes and xanthophylls in the two branched pathways. It seems that the optimal N application of 250 kg ha^-1^ would favor the fast transformation of (E/Z)-phytoene and phytofluene into capsanthin and capsorubin via a large increase in the accumulation of zeaxanthin and violaxanthin but limiting the production of neoxanthin in the γ-carotene pathway, with reduced carbon influx into the δ-carotene pathway. However, the higher N application rate of 400 kg N ha^-1^ tends to maintain greater accumulation of precursors, (E/Z)-phytoene and phytofluene, and increases downstream production of ϵ-carotene, α-carotene, and α-cryptoxanthin in the δ-carotene pathway and neoxanthin in the γ-carotene pathway, but with relatively reduced capsanthin and capsorubin production. Thus, although the total carotenoid content was similar under N250 and N400 conditions, it can be speculated that the optimal N supply (250 kg N ha^-1^) is more beneficial for *de novo* carotenoid biosynthesis into capsanthin and capsorubin.

As mentioned above, four catalytic enzymes (PSY, LCYB, LCYE, and CCS) play important roles in carotenoid synthesis. A previous study has reported positive correlations between total carotenoid content and *PSY1*, *LCYB*, and *CCS* expression in pepper fruit pulp, and *CCS* expression in the fruit peel (Filyushin et al., 2020). Furthermore, another combined metabolome and transcriptome analysis of pepper fruits with different colors has found that the *PSY1* gene regulates the accumulation of phytoene, the *LCYB* and *LCYE* genes synergistically regulate the accumulation of α-carotene, γ-carotene, and β-carotene, while the accumulation of capsanthin was not determined solely by the *CCS* gene (Liu et al., 2020). Moreover, a recent work reported an R-R-type MYB transcription factor promoting carotenoid biosynthetic gene transcript levels and capsanthin content (Song et al., 2023). These studies provide additional insights into the potential genetic regulation of carotenoid biosynthesis, but the role of N nutrition on the expression of these genes in pepper fruits requires further study.

### 4.3 Phenolic compound accumulation and their relationships with carotenoids as influenced by N supply

Phenolic acids and flavonoids are the major groups of phenolic compounds present in pepper fruits (Suseela and Tharayil, 2018). Previous studies have shown that p-coumaric, caffeic, sinapic, and ferulic glycosides are the characteristic phenolic acid derivatives in pepper fruits (Materska and Perucka, 2005; Baenas et al., 2019). Research suggests that N fertilization decreases the amount of polyphenols in some plants (Larbat et al., 2012; Radusiene et al., 2019; Narvekar and Tharayil, 2021). In this study, N supply (N250 and N400) dramatically decreased the total phenol content (**Table 1**), in accordance with former reports. Most of the phenolic compounds are biosynthesized through the phenylpropanoid pathway which starts with phenylalanine (Jakovljevic et al., 2019). The restriction of N will decrease protein synthesis and thus reduce competition for phenylalanine (Bustamante et al., 2020). In the present study, caffeic aldehyde was upregulated under N deficiency (N0) (**Figure 6**). However, the metabolites in the other branches of phenylpropane metabolism (scopolin and sinapoyl malate) were not affected, which may be due to the reorientation of metabolic fluxes between branches of the phenylpropane pathway to cope with low N stress and maintain the homeostasis of metabolism (Dong and Lin, 2021).

Flavonoids are synthesized from phenylalanine via phenylpropanoid and flavonoid pathways (Tohge et al., 2017; Liu et al., 2020). Previous studies have found that expression of flavonoid biosynthesis pathway genes is affected by nutrient depletion (Liu et al., 2021). N deficiency could decrease chlorophyll content and photosynthesis (Li et al., 2021). A malfunctioning photosystem renders the plants more exposed to oxidative damage and the increased flavanoid production in such conditions is thought to protect plants from photo-oxidative damage (Gill and Tuteja, 2010). In this study, significant enrichment was observed for metabolites in flavonoid biosynthesis and flavone-flavonol biosynthesis under the control (N0) condition (**Figure 6**). Moreover, N application rate was negatively correlated with most flavonoid compounds, especially luteolin, kaempferol, eriodictyol, hesperetin, and quercetin derivatives, which were present either at the C-3 or C-7 position in the form of O-glycosides, as well as naringenin and naringenin chalcone (**Supplementary Table S7**). Under the influence of flavonol synthase, dihydroflavonol can be synthesized into flavonols such as kaempferol, quercetin, and myricetin (Shen et al., 2022). Therefore, the downregulation of kaempferol-3-O-rutinoside (nicotiflorin) and quercetin-3-O-(2″-O-Xylosyl) rutinoside observed in this study might have caused higher dihydromyricetin accumulation under the N250 and N400 conditions compared with the N0 condition (**Figure 6**). These results indicate that sufficient N supply may reduce flavonoid accumulation and their contribution to the red pigmentation of pepper fruit.

The synthesis and accumulation of carotenoids usually accompany the degradation of chlorophyll and flavonoids when pepper fruits mature (Liu et al., 2020). Both carotenoids and flavonoids are carbon-based compounds that do not contain N, but they may demonstrate contrasting responses to N deficiency (Becker et al., 2015). In the present study, correlation network analysis showed that most carotenoids were negatively correlated with flavonoids and phenolic acids (**Figure 7**). N application rates showed a negative correlation with most flavonoids and phenolic acids, but a positive correlation with most carotenoids, lignans, and coumarins (**Supplementary Table S7**). These results partially confirmed the common viewpoint that high N supply could decrease secondary metabolite accumulation in plants (Deng et al., 2019). Although there was a general decrease in the relative amount of most flavonoids and phenolic acids with increased N supply, the production of other compounds were stimulated, or not affected (**Supplementary Figure S6**; **Supplementary Table S6**), as shown in other studies (Ormeno and Fernandez, 2012; Saloner and Bernstein, 2021). It is worth noting that content of some flavonoids and phenolic acids in sub-class 3 and 5 peaked under N250 compared with N0 and N400, indicating the optimized N supply was beneficial for these compounds. Thus, our results highlight the differential response of secondary metabolites to N supply in pepper fruits, and the consequent variation in pigments content, composition, and accumulation. Understanding the mechanistic basis of this biochemical response of pepper fruit will help develop molecular and crop management tools and technologies for producing nutritionally-superior peppers.

## 5 Conclusions

In this study, growth and color parameters together with targeted and non-targeted metabolomics of secondary products were used to understand and explain the color formation in mature pepper fruits produced under different N supply. N fertilization promoted carotenoid biosynthesis but downregulated phenylpropanoid and flavonoid biosynthesis in peppers. The red color deepened with increased N supply, which is attributed to the accumulation of carotenoids (mainly capsanthin, zeaxanthin, and capsorubin), as well as the decrease in flavonoids (especially luteolin, kaempferol, eriodictyol, hesperetin, and quercetin derivatives) and phenolic acids (caffeic aldehyde). Compared with the current practice of farmers (400 kg N ha^-1^), a much-reduced N input (250 kg N ha^-1^) than is sufficient to achieve high yield and high pepper fruit nutritional quality for human health, in addition to promoting the red color formation that increases consumer appeal. Furthermore, the reduced N input reduces production cost and facilitates positive environmental outcomes, i.e., reducing N leaching loss. Future work can be combined with transcriptomics, proteomics, and other molecular methods to further reveal the regulatory mechanism of N nutrition on pigment formation in peppers.

## Data availability statement

The original contributions presented in the study are included in the article/**Supplementary Material**. Further inquiries can be directed to the corresponding authors.

## Author contributions

LZ: Conceptualization, Data curation, Methodology, Writing – original draft. FZ: Methodology, Writing – review & editing. XH: Methodology, Writing – review & editing. YD: Methodology, Writing – review & editing. KS: Methodology, Writing – review & editing. SL: Methodology, Writing – review & editing. XW: Writing – review & editing. HY: Writing – review & editing. WZ: Writing – review & editing. PL: Writing – review & editing. XC: Conceptualization, Funding acquisition, Supervision, Writing – review & editing. YD: Conceptualization, Supervision, Writing – original draft.
