# Supplementary material for: Comparative metabolomics reveals complex metabolic shifts associated with nitrogen-induced color development in mature pepper fruit
Source: Front Plant Sci. 2024 Feb 20;15:1319680. doi: 10.3389/fpls.2024.1319680 (PMC10912300; doi:10.3389/fpls.2024.1319680)
Supplement: Supplementary file 1 [file DataSheet_1.docx]

**Supplementary Method 1. (Methods S1)** UPLC and APCI-MS/MS conditions for targeted carotenoids.

The sample extracts were analyzed using an UPLC-APCI-MS/MS system (UPLC， ExionLC™ AD，https://sciex.com.cn/ ; MS，Applied Biosystems 6500 Triple Quadrupole, https://sciex.com.cn/ ). The analytical conditions were as follow, LC: column, YMC C30(3 μm, 100 mm×2.0 mm i.d); solvent system, methanol：acetonitrile (1:3, v/v) with 0.01% BHT and 0.1% formic acid (A), methyl tert-butyl ether with 0.01% BHT (B); gradient program, started at 0% B (0-3 min), increased to 70% B (3-5 min), then increased to 95% B (5-9 min), finaly ramped back to 0% B (10-11 min); flow rate, 0.8 mL/min; temperature, 28°C; injection volume: 2 μL.

Linear ion trap (LIT) and triple quadrupole (QQQ) scans were acquired on a triple quadrupole-linear ion trap mass spectrometer (QTRAP), QTRAP® 6500+ LC-MS/MS System, equipped with an APCI Heated Nebulizer, operating in positive ion mode and controlled by Analyst 1.6.3 software (Sciex). The APCI source operation parameters were as follows: ion source, APCI+; source temperature 350°C; curtain gas (CUR) 25.0 psi. Carotenoids were analyzed using scheduled multiple reaction monitoring (MRM). Data acquisitions were performed using Analyst 1.6.3 software (Sciex). Multiquant 3.0.3 software (Sciex) was used to quantify all metabolites. Mass spectrometer parameters including the declustering potentials (DP) and collision energies (CE) for individual MRM transitions were done with further DP and CE optimization. A specific set of MRM transitions were monitored for each period according to the metabolites eluted within this period.

**Supplementary Method 1. (Methods S2)** UPLC and ESI-Q TRAP-MS/MS conditions for non-targeted metabolites

The sample extracts were analyzed using an UPLC-ESI-MS/MS system (UPLC, SHIMADZU Nexera X2, https://www.shimadzu.com.cn/; MS, Applied Biosystems 4500 Q TRAP, https://www.thermofisher.cn/cn/zh/home/brands/applied- biosystems. html). The analytical conditions were as follows, UPLC: column, Agilent SB-C18 (1.8 µm, 2.1 mm * 100 mm); The mobile phase was consisted of solvent A, pure water with 0.1% formic acid, and solvent B, acetonitrile with 0.1% formic acid. Sample measurements were performed with a gradient program that employed the starting conditions of 95% A, 5% B. Within 9 min, a linear gradient to 5% A, 95% B was programmed, and a composition of 5% A, 95% B was kept for 1 min. Subsequently, a composition of 95% A, 5.0% B was adjusted within 1.1 min and kept for 2.9 min. The flow velocity was set as 0.35 mL per minute; The column oven was set to 40°C; The injection volume was 4 μL. The effluent was alternatively connected to an ESI-triple quadrupole-linear ion trap (QTRAP)-MS.

LIT and triple quadrupole (QQQ) scans were acquired on a triple quadrupole-linear ion trap mass spectrometer (Q TRAP), AB4500 Q TRAP UPLC/MS/MS System, equipped with an ESI Turbo Ion-Spray interface, operating in positive and negative ion mode and controlled by Analyst 1.6.3 software (AB Sciex). The ESI source operation parameters were as follows: ion source, turbo spray; source temperature 550°C; ion spray voltage (IS) 5500 V (positive ion mode)/-4500 V (negative ion mode); ion source gas I (GSI), gas II(GSII), curtain gas (CUR) set at 50, 60, and 25.0 psi, respectively; the collision-activated dissociation (CAD) was high. Instrument tuning and mass calibration were performed with 10 and 100 μmol/L polypropylene glycol solutions in QQQ and LIT modes, respectively. QQQ scans were acquired as MRM experiments with collision gas (nitrogen) set to medium. DP and CE for individual MRM transitions was done with further DP and CE optimization. A specific set of MRM transitions were monitored for each period according to the metabolites eluted within this period.
