# Supplementary material for: Comparative metabolomics reveals complex metabolic shifts associated with nitrogen-induced color development in mature pepper fruit
Source: Front Plant Sci. 2024 Feb 20;15:1319680. doi: 10.3389/fpls.2024.1319680 (PMC10912300; doi:10.3389/fpls.2024.1319680)
Supplement: Supplementary file 2 [file DataSheet_2.docx]

***Supplementary Figures***


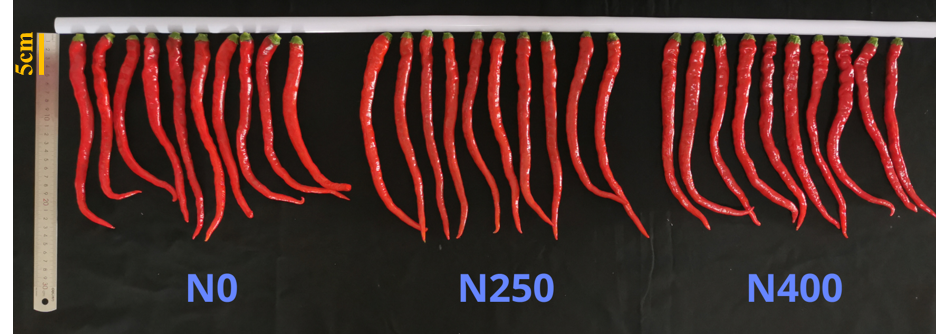


**Figure S1**. Pepper fruit at the mature red stage.


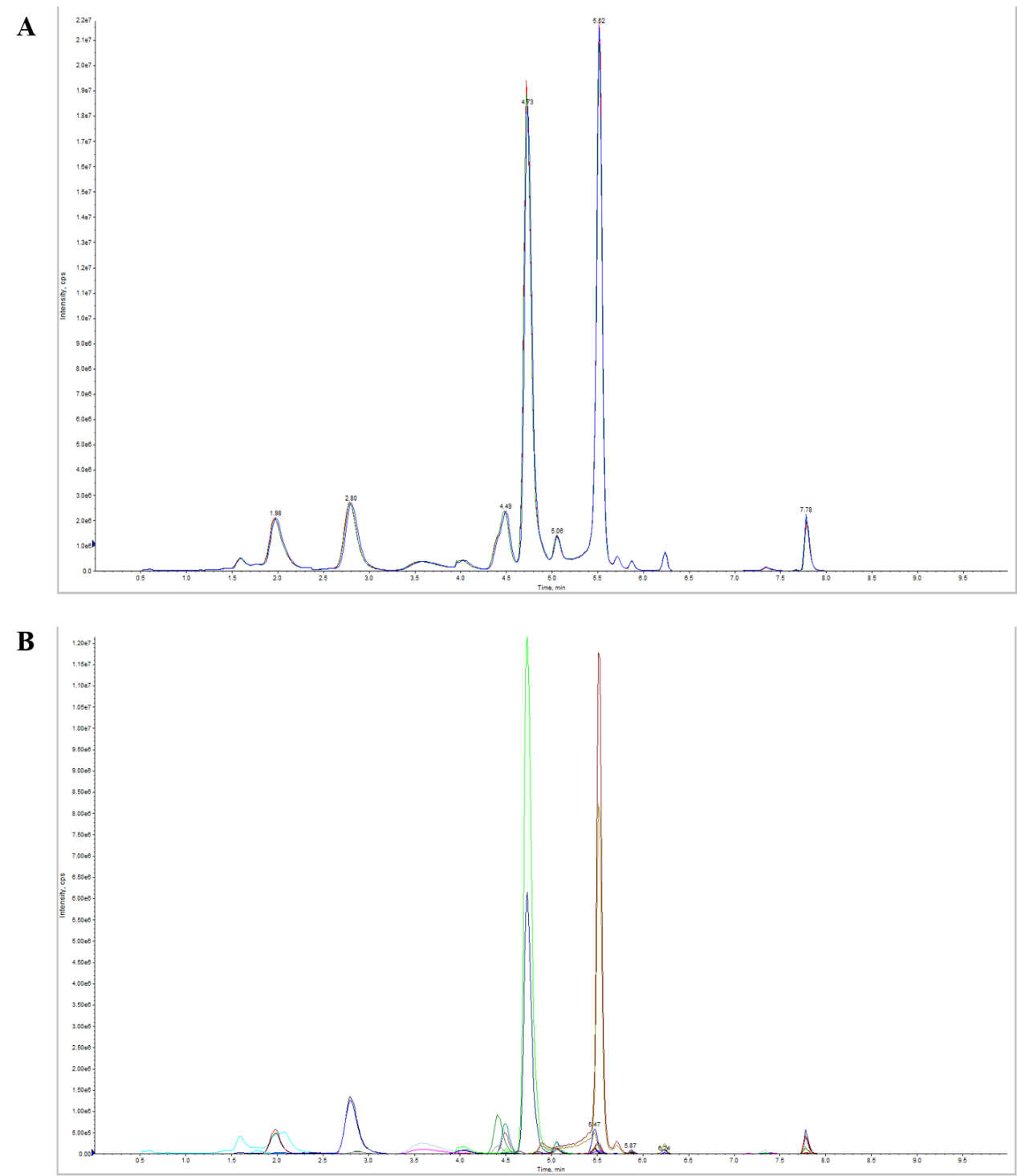


**Figure S2**. Representative total ion chromatogram of a quality control sample (**A**) and multipeak mass spectral chromatogram of metabolites acquired in multiple reaction monitoring mode (**B**). The abscissa is retention time (RT) of carotenoid detection, and the ordinate is ion counts per second (CPS).


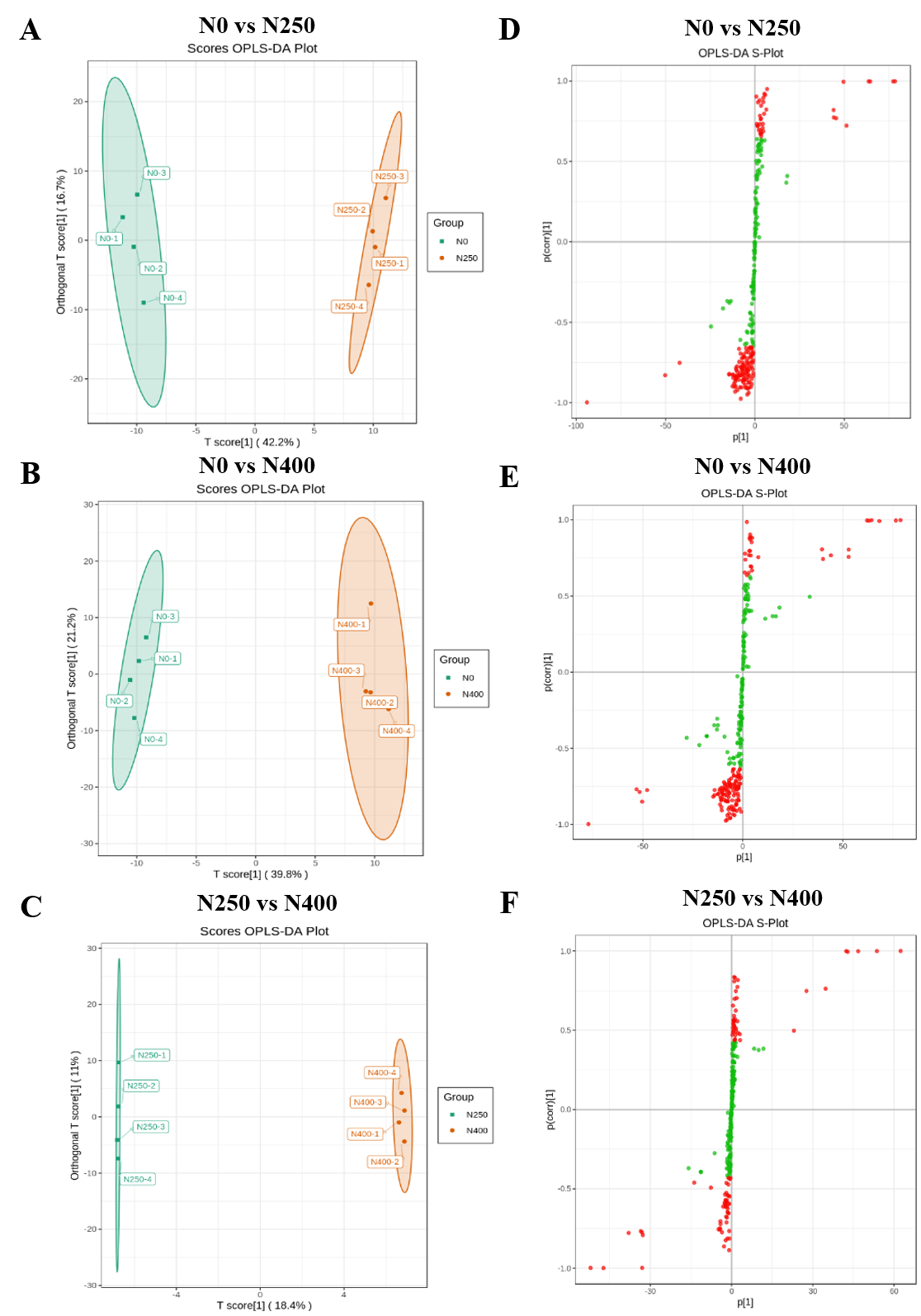


**Figure S3**. Orthogonal partial least squares-discriminant analysis (OPLS-DA) score (**A**, **B**, **C**) and corresponding OPLS-DA loading S-plot (**D**, **E**, **F**) of compared samples in N0 vs. N250, N0 vs. N400, and N250 vs. N400.


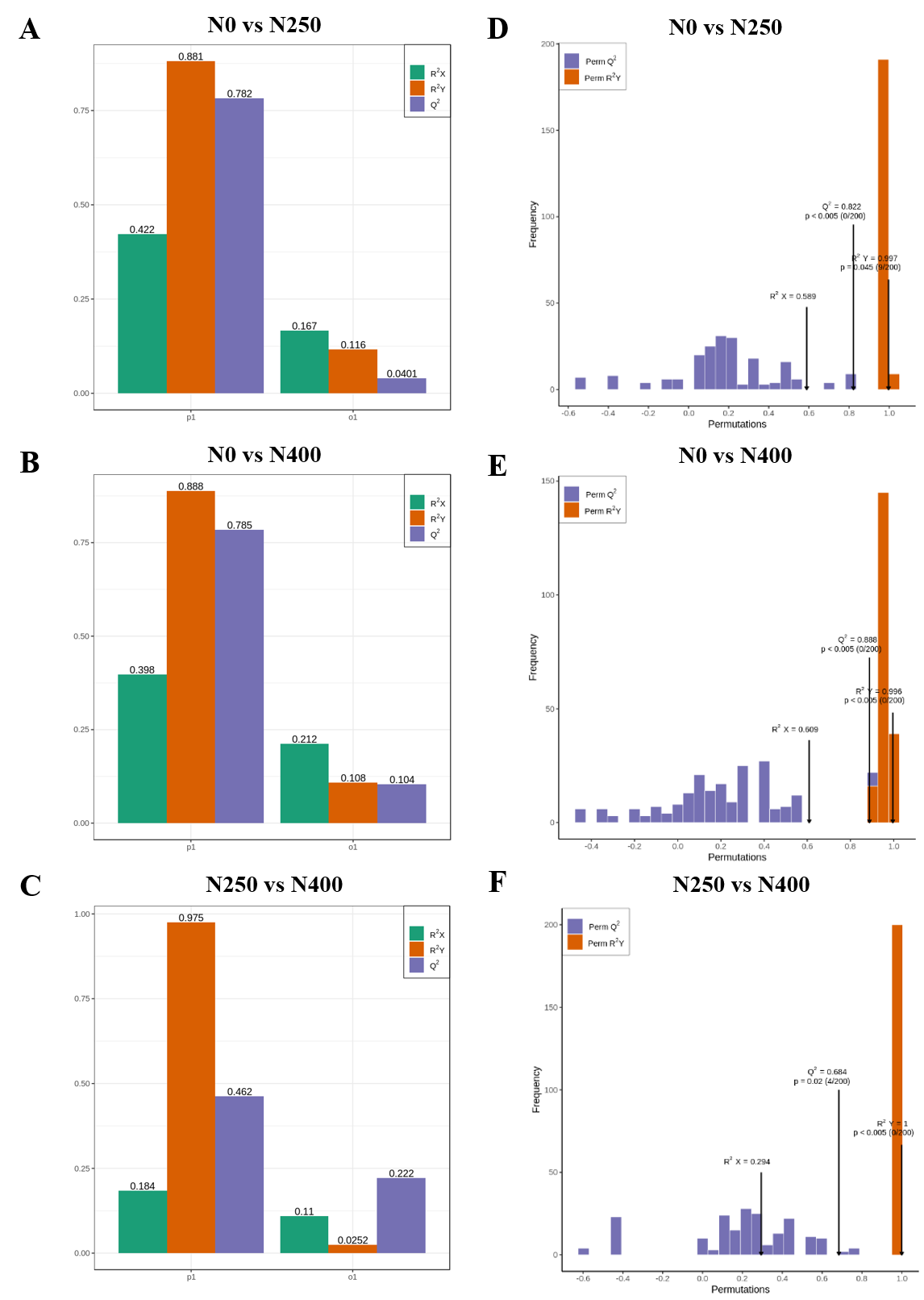


**Figure S4**. Orthogonal partial least squares-discriminant analysis (OPLS-DA) model (**A**, **B**, **C**) and permutation test plot with 200 iterations (**D**, **E**, **F**) of compared samples in N0 vs. N250, N0 vs. N400, and N250 vs. N400.


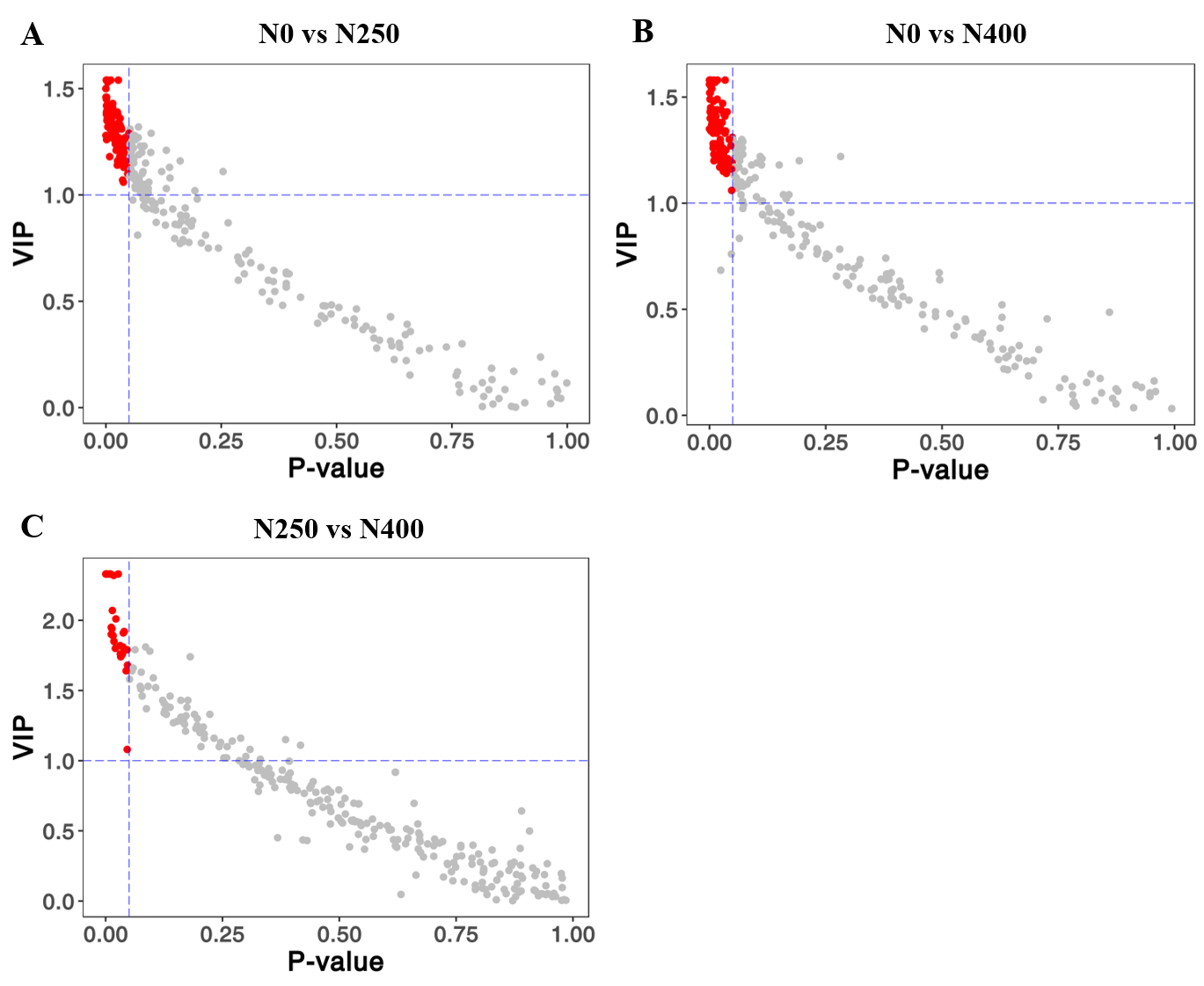


**Figure S5**. The relationship between variable importance in projection (VIP) values and *P*-values for comparison of N0 vs. N250 (**A**), N0 vs. N400 (**B**), and N250 vs. N400 (**C**). The red dots represent differentially accumulated metabolites (DAMs) between N treatments screened by VIP ≥ 1 and *P* < 0.05 (t-test).


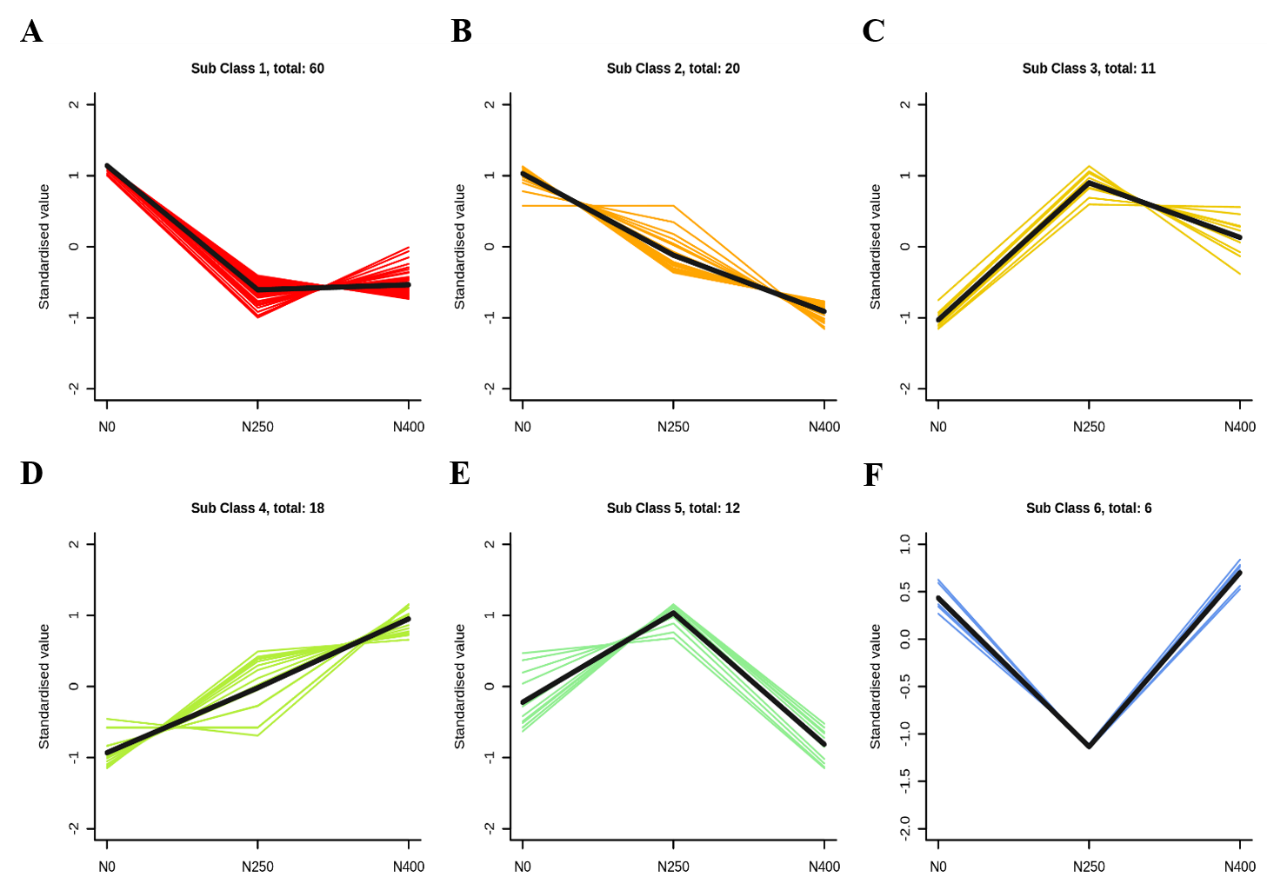


**Figure S6**. The K-means analysis of secondary metabolites detected in the pericarp of pepper fruit between pairs of N treatments at the mature red stage. The Z-score was standardized for the average value of relative content of differential metabolites in each group, and K-means clustering analysis is performed.
